# MRI parameters predict central lumbar spinal stenosis combined with redundant nerve roots: a prospective MRI study

**DOI:** 10.3389/fneur.2024.1385770

**Published:** 2024-05-27

**Authors:** Jingli Qian, Kaiwen Liang, Xianping Luo, Caiyun Ying

**Affiliations:** ^1^Department of Traditional Chinese Medicine and Rehabilitation, People's Hospital of Chongqing Liangjiang New Area, Chongqing, China; ^2^Department of Health Management Center, The First Affiliated Hospital of Chongqing Medical University, Chongqing, China; ^3^Department of Radiology, People's Hospital of Chongqing Liangjiang New Area, Chongqing, China

**Keywords:** lumbar spinal stenosis (LSS), redundant nerve roots (RNRs), magnetic resonance imaging (MRI), epidural fat, risk factors

## Abstract

**Background:**

To observe changes in the cauda equina nerve on lumbar MRI in patients with central lumbar spinal stenosis (LSS).

**Methods:**

878 patients diagnosed with LSS by clinical and MRI were divided into the redundant group (204 patients) and the nonredundant group (674 patients) according to the presence or absence of redundant nerve roots (RNRs). The anteroposterior diameter of the spinal canal (APDS) and the presence of multiple level stenosis, disc herniation, thickening of ligamentum flavum (LF) and increased epidural fat were assessed on MRI. Univariate and multivariate logistic regression analyses were performed to explore the predictors of LSS combined with RNRs.

**Results:**

Patients with LSS combined with RNRs had thicker epidural fat, smaller APDS and more combined multifaceted stenosis. Female patients and older LSS patients were more likely to develop RNRs; there was no difference between two groups in terms of disc herniation (*p* > 0. 05). Age, APDS, multiple level stenosis, and increased epidural fat were significantly correlated with the formation of LSS combined with RNRs (*p* < 0.05).

**Conclusion:**

A smaller APDS and the presence of multiple level stenosis, thickening of LF, and increased epidural fat may be manifestations of anatomical differences in patients with LSS combined with RNRs. Age, APDS, multiple level stenosis, and increased epidural fat play important roles. The lumbar spine was measured and its anatomy was observed using multiple methods, and cauda equina changes were assessed to identify the best anatomical predictors and provide new therapeutic strategies for the management of LSS combined with RNRs.

## 1 Introduction

Redundant nerve roots (RNRs) of the cauda equina appear dilated, curved and entangled in the spinal canal. Such a phenomenon was first identified and described by Verbiest (1) in 1954 during X-ray myelography. The sign was named RNRs by Cressmen and Pawl (2) in 1968. One of the main reasons for performing lumbar spine surgery for lumbar spinal stenosis (LSS) was that 26% of the population over 65 years of age (3) had LSS and that 33.8% ~ 42.3% (4–7) of these patients had comorbid RNRs. These patients tend to have clinically typical neurological symptoms, including neurogenic intermittent claudication, pain in the lower limbs and lower back, a longer duration of clinical symptoms and poor quality of life, thus requiring surgical relief. Currently, surgery for patients with LSS mainly targets the responsible segment to remove all compression-causing materials, including hyperplastic bone, intervertebral discs, and ligamentum flavum, to free the nerve root through adequate decompression and to alleviate the patient’s nerve compression symptoms. For patients with LSS combined with RNRs, it is important to not only adequately decompress the nerve roots intraoperatively but to also assess whether the RNRs can be released, i.e., the dura mater needs to be opened, and the entangled and knotted cauda equina nerves need to be loosened to achieve a better outcome. It has been concluded that LSS patients with combined RNRs have worse preoperative scores than LSS patients without RNRs (8, 9), as well as worse postoperative clinical scores and lower cure rates (4, 10, 11), which is one of the reasons for the poor prognosis of the patients. Some scholars believe (12, 13) that patients with LSS should undergo spinal canal surgical decompression as early as possible before irreversible damage to the cauda equina occurs to release the RNRs and achieve better reduction of nerve compression symptoms. Therefore, for LSS patients, early and accurate diagnosis of RNRs becomes the key to improving surgical efficacy.

**Figure 1:**
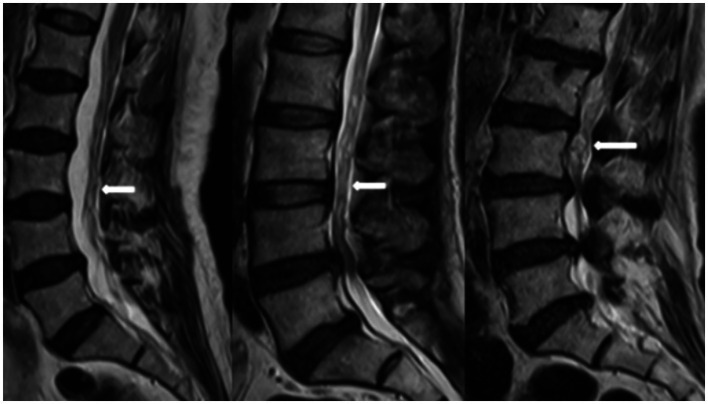
RNRs show thickening, buckling, and serpentine- or loop-shaped on their T2-weighted sagittal MRI slices.

Magnetic resonance imaging (MRI) technology has become a reliable imaging method for the diagnosis of RNRs and LSS due to its good soft tissue resolution, multiparameter measurement, and multisequence and multiplane imaging methods, making it a key player in surgical decision-making. On preoperative magnetic resonance imaging, RNRs show thickening, buckling, and serpentine- or loop-shaped on their T2-weighted sagittal MRI slices (Figure 1), which are often further diagnosed on the basis of a diagnosis of central LSS (14). Magnetic resonance imaging of central lumbar LSS shows a median sagittal diameter of the lumbar spinal canal that is less than 15 mm. The main imaging signs include vertebral body and vertebral small joint osteophytes, vertebral body slippage and instability, disc bulging, disc herniation or calcification due to degeneration and degeneration of the intervertebral disc, and hypertrophy or calcification of the ligamentum flavum. The above imaging method facilitates comprehensive analysis of the vertebral body itself and the surrounding tissue anatomy, and the epidural fat is an important soft tissue that maintains the vertebral body structure and movement. Tissue imaging study of other surrounding organs has received increasing attention. For example, perirenal fat thickness (PFT) is highly correlated with renal trauma grade (15), but it is often neglected in the imaging diagnosis of LSS patients and has not been reported. In magnetic resonance imaging studies of LSS combined with RNRs (6, 13, 16–18), it was shown that the sagittal and transverse diameters of the spinal canal at the level of maximal stenosis, the area of the dural sac, the thickness of the ligamentum flavum, and the presence or absence of disc herniation and lumbar spondylolisthesis contributed to the development of RNRs, and it was concluded that for female patients, the older they were, and the higher the degree of lumbar canal stenosis, the more likely they were to develop RNRs. Studies evaluating cause and occurrence have shown that patients with thickening and hypertrophy of the ligamentum flavum and lumbar spondylolisthesis have a higher chance of developing RNRs. However, the studies are not comprehensive; for example, the correlation between the condition of epidural fat and RNRs has not been reported, while the early diagnosis of RNRs, i.e., the use of different causes in predicting the occurrence of RNRs, has not been reported.

In this study, on the one hand, it is important to comprehensively assess the causes of the formation of LSS combined with RNRs, especially the distribution of epidural fat, through preoperative magnetic resonance imaging to accurately diagnose and implement intraoperative targeted treatment, and on the other hand, through the prediction of the occurrence of RNRs to achieve early diagnosis, i.e., early diagnosis and early treatment, and to provide an imaging basis for improving the clinical cure rate.

## 2 Materials and methods

### 2.1 Subjects

The study was approved by the Medical Ethics Committee of the People’s Hospital of Chongqing Liangjiang New Area (No: 27). The committee waived the need for informed consent because of the observational nature of this study. A total of 3,027 patients who underwent MRI examination in our hospital from January 2022 to March 2023 were prospectively enrolled. The inclusion criteria were as follows: ① The presence of clinical symptoms of spinal stenosis, such as neurogenic claudication or pain radiating bilaterally to the lower limbs. ② LSS was diagnosed by clinical and MRI examinations. The exclusion criteria were as follows: ① Degenerative lumbar spondylolisthesis, with a slip ≥3 mm verified in lateral view. ② Presence of a lumbosacral scoliosis of more than 20 degrees, verified on AP-view. ③ Patients with a history of trauma fracture, spinal infection, tumor and surgery. ④ Acute traumatized patients and patients with motion artifacts during scanning due to intolerance of pain, which seriously affects image quality.

### 2.2 MRI imaging

A SIEMENS 1.5 T magnetic resonance imaging scanner was used, with a spinal matrix coil selected, and the subject was placed in a supine position with the lower limbs straightened and head elevated, which is the conventional position for magnetic resonance imaging of the lumbar spine. The scanning range included the upper edge of the T12 vertebra to the level of the lower edge of the S2 vertebra. The lumbar spine MRI scanning sequences and scanning parameters were set as follows: sagittal T2WI (repetition time (TR) of 2,800 ms, echo time (TE) of 89 ms, matrix of 320 × 240), sagittal T1WI (TR of 645 ms, TE of 11 ms, matrix of 320 × 256), and sagittal fat-suppressed T2WI sequence (TR of 3,800 ms, TE of 70 ms, matrix of 256 × 192). The above sequences were performed with a layer thickness of 4 mm, layer spacing of 0.1 mm, and a field of view (FOV) of 300 mm × 300 mm; cross-sectional T2WI (TR of 2,900 ms, TE of 83 ms, matrix of 320 × 224) was performed with a layer thickness of 4 mm, layer spacing of 0.1 mm, and FOV of 300 mm × 300 mm.

### 2.3 Measurement and observation of relevant indicators

This included measurement of APDS at the stenosis, thickness of the ligamentum flavum, observation of disc herniation, lumbar epidural fat in magnetic resonance images for sagittal grading and cross-sectional classification, and other parameters.

#### 2.3.1 APDS

**Figure 2:**
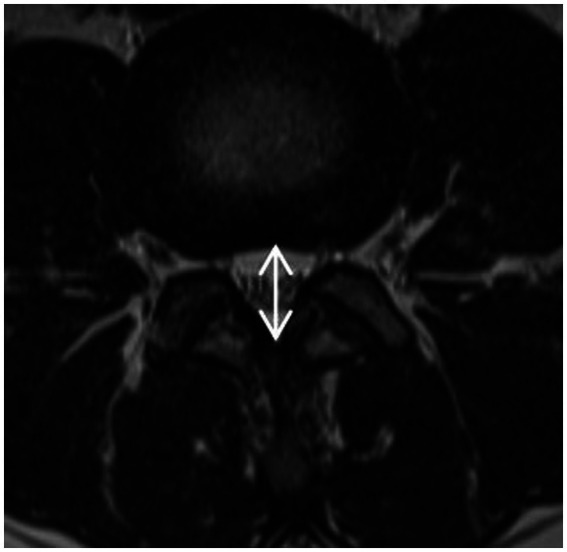
APDS: It was measured at the disc level by drawing a line between the posterior border of discus and the ligamentum flavum at midline.

The narrowest level was selected at the disc level between L1-S1 of the patient. It was measured at the disc level by drawing a line between the posterior border of the discus and the ligamentum flavum at midline (14) (Figure 2).

#### 2.3.2 Measurement of the thickness of the ligamentum flavum

**Figure 3:**
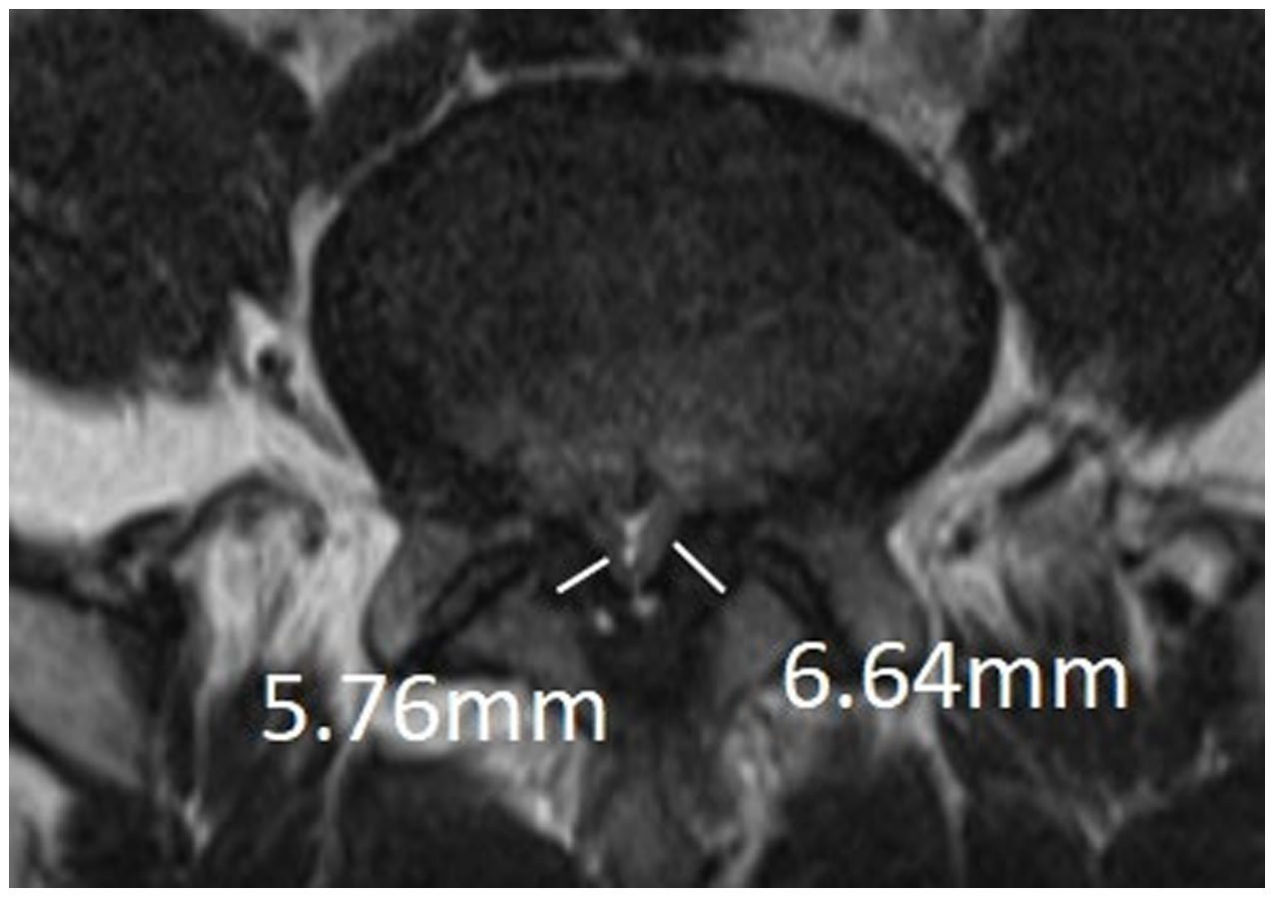
Measurement of thickness of ligamentum flavum: The measurement is at the midpoint of the medial-lateral line of the ligamentum flavum and perpendicular to its long axis.

The measurement is at the midpoint of the medial-lateral line of the ligamentum flavum and perpendicular to its long axis (Figure 3). The ligamentum flavum hypertrophy group was defined as 4.0 mm or more on either side of the ligamentum flavum bilaterally, and the nonthickening group was defined as less than 4.0 mm on both sides.

#### 2.3.3 Lumbar epidural fat

Ishikawa et al. (19) classified epidural fat in MRI in both cross-sectional and sagittal planes. Morphological characteristics of epidural fat recognisable on MR images from L1-2 to L5-S1 were assessed using the lumbar SEL grading system. These classifications and gradings represent the presence and severity of epidural fat compression of the dural sac.

#### 2.3.4 The cross-sectional classification of epidural fat

**Figure 4:**
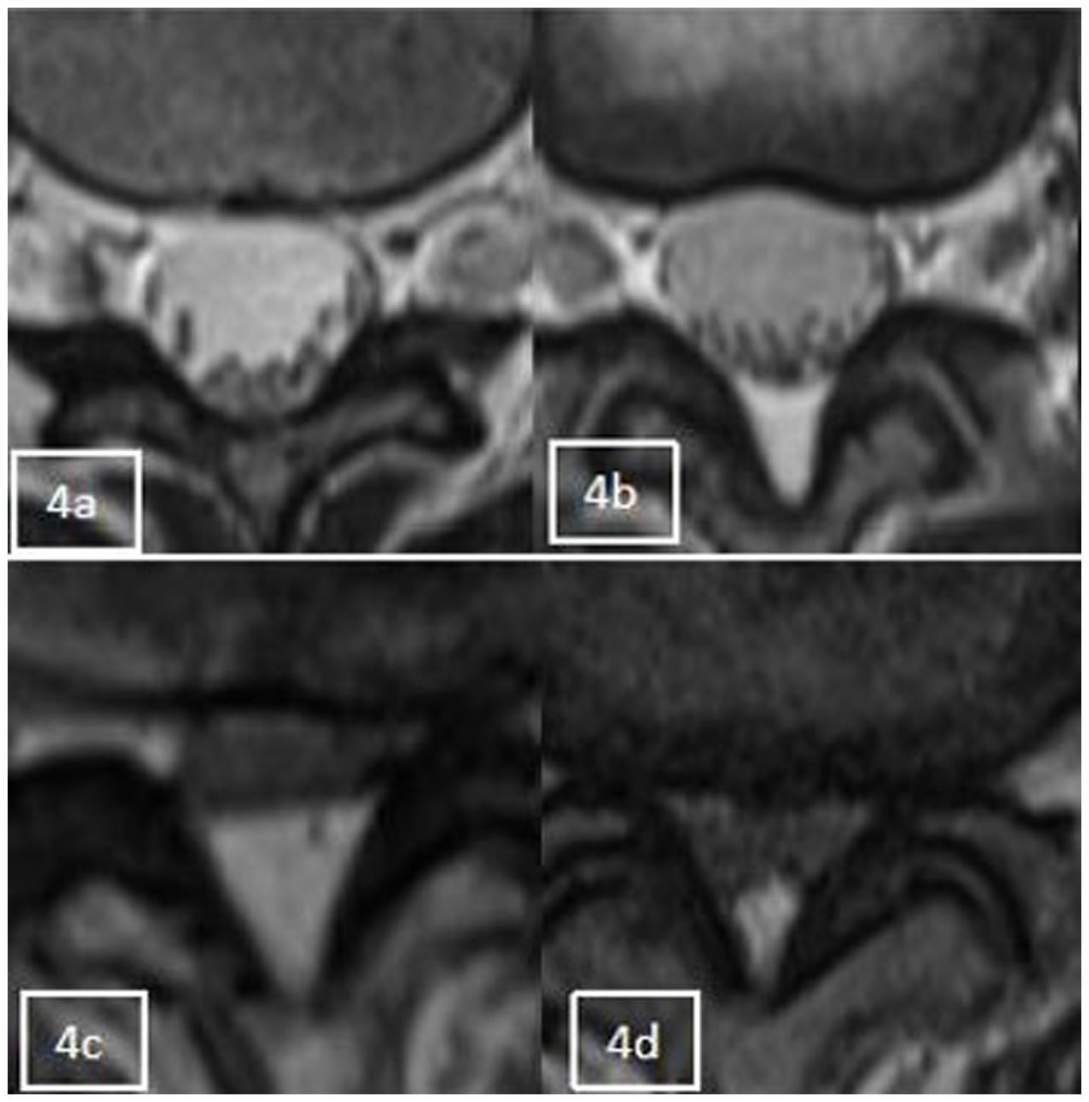
Axial appearance of epidural fat on T2-weighted MR images. Fat accumulation at each intervertebral disc level from L1–2 to L4–5 was classified as follows: absent **(A)**, concave **(B)**, flat **(C)**, or convex **(D)**.

**Figure 5:**
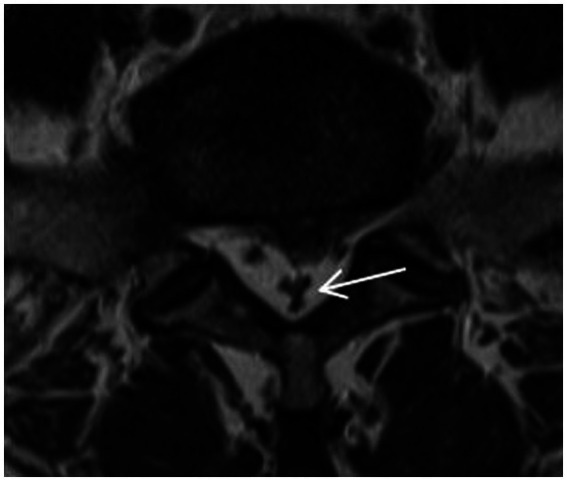
The “Y” sign: when epidural fat was severely accumulated in the spinal canal, it compressed the dural sac into a Y-shape and named it the “Y” sign.

Categories 0–3, as shown in Figure 4, and according to whether the dural sac was deformed by compression or not, it was divided into Group A without deformation by compression (category 0 + 1) and Group B with deformation by compression (category 2 + 3). When a segment showed the “Y” sign, it was identified as category 3 in the cross-sectional classification in this study. Kuhn et al. (20) found that when epidural fat was severely accumulated in the spinal canal, it compressed the dural sac into a Y-shape and named it the “Y” sign, as shown in Figure 5.

#### 2.3.5 Sagittal grading of epidural fat

**Figure 6:**
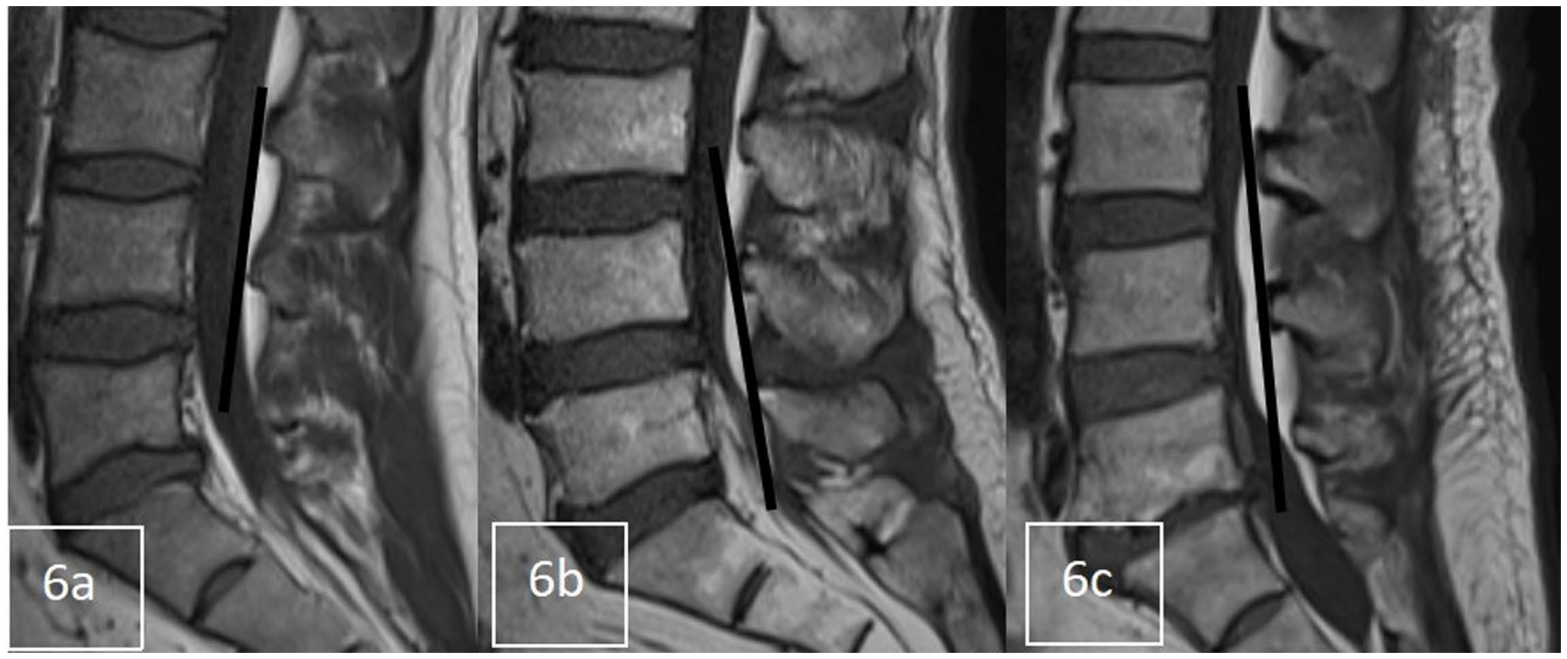
Sagittal grading of epidural fat on T1-weighted MR images. **(A)** Grade 1 was defined as epidural fat observed within the border between the anterosuperior edges of the upper and lower neighboring neural arches. **(B)** Grade 2 was defined as fat observed over the border at the middle but not at the edges of neural arches on both sides. **(C)** Grade 3 was defined as fat observed over the border at the edges of neural arches on at least one side.

Grades 1–3, as shown in Figure 6, and according to whether the dural sac was deformed by compression or not, was divided into Group C (Grade 1) without compression deformation and Group D (Grade 2 + Grade 3) with compression deformation.

### 2.4 Statistical analysis

Data were analyzed using SPSS Statistics 26.0. Data for continuous variables were expressed as the mean standard deviation ( $\bar{x}\pm s$ ) and compared using *t* test; data for categorical variables were expressed as frequency (percentage) [*n* (%)], and unordered categorical variables were compared using the chi-square test or Fisher’s exact test; ordered categorical variables were compared using the Mann–Whitney U test; statistically significant factors in the univariate analyses were extracted, and statistically significant factors in the multivariate analyses were used using binary logistic regression analysis for multifactorial analysis; *p* < 0.05 was used to indicate statistically significant differences.

## 3 Results

Before the start of the experiment, a diagnostic imaging deputy chief physician trained three attending physicians. All four physicians had over 15 years of clinical experience, and the measurement method was unified. The above measurement data were independently evaluated and measured by the three physicians on the PACS system, and the average of the measured values was taken for statistical analysis. In those patients with LSS on lumbar MRI, the presence of RNRs was evaluated with consensus by the three physicians. The results were reviewed again by a diagnostic imaging deputy chief physician, and the four physicians negotiated to solve the inconsistency of the results.

A total of 878 patients with central LSS who met the clinical and imaging criteria were screened according to the nadir criteria, including 356 (40.5%) males and 522 (59.4%) females, with an age range of 15–92 years old and a mean age of 52.61 ± 15.165 years. They were divided into redundant and nonredundant groups according to the presence or absence of RNRs, with a total of 204 cases (32.2%) in the RNR group, 67 (32.8%) males and 137 (67.2%) females, with a mean age of 64.34 ± 12.426 years; and a total of 674 cases (76.8%) in the nonredundant group, 289 (42.9%) males and 385 (57.1%) females, with a mean age of 49.06 ± 14.098 years.

### 3.1 Univariate analysis

**Figure 7:**
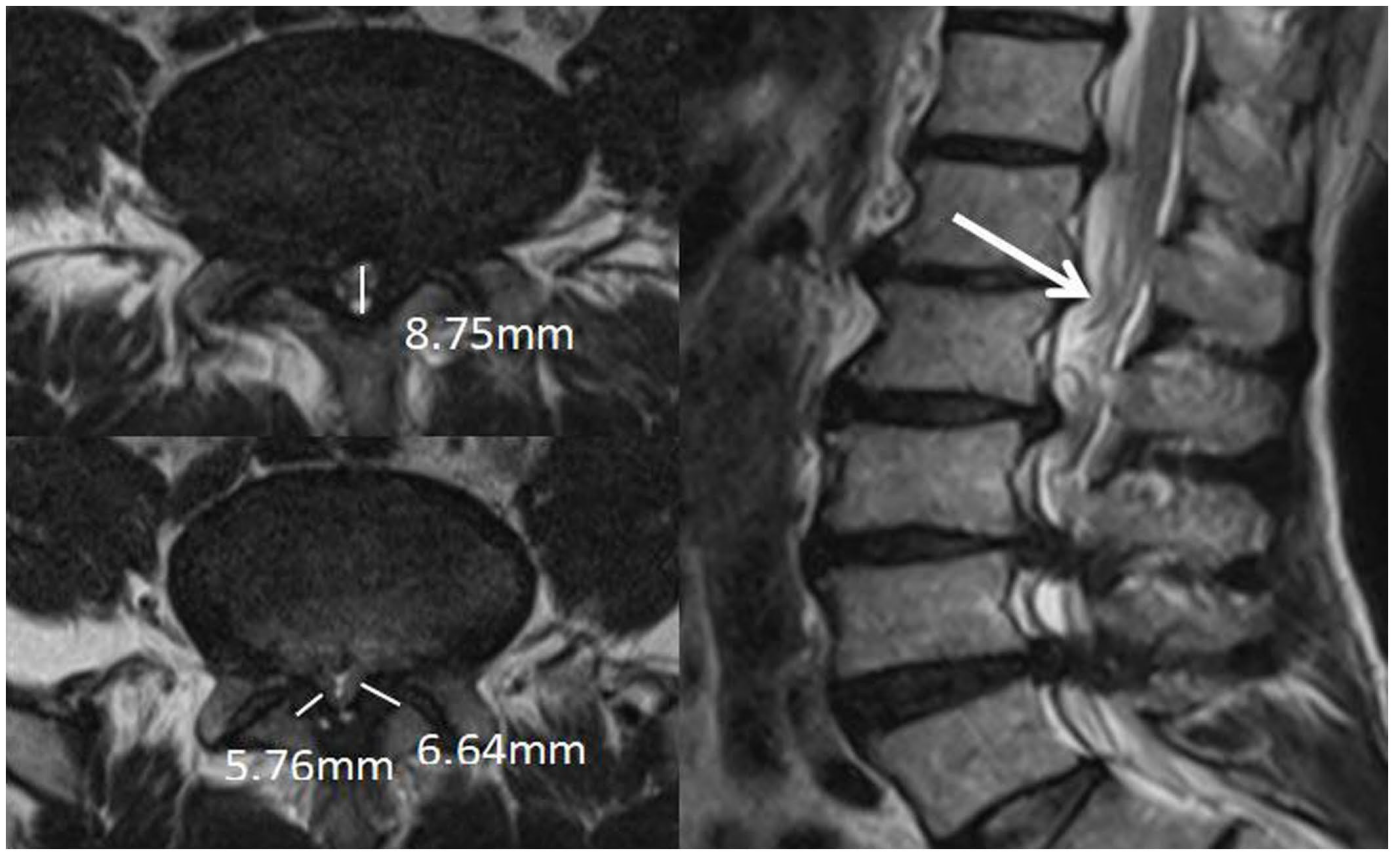
Patient, male, 67 years old, with recurrent low back pain for 2 years, recurrence and exacerbation for 3 months, lumbosacral pressure and percussion. Magnetic resonance imaging: bilateral ligamentum flavum hypertrophy and spinal stenosis, and the cauda equina nerve in the sagittal position at T2WI showed flaccid, tortuous, and entangled high-signal shadows.

**Figure 8:**
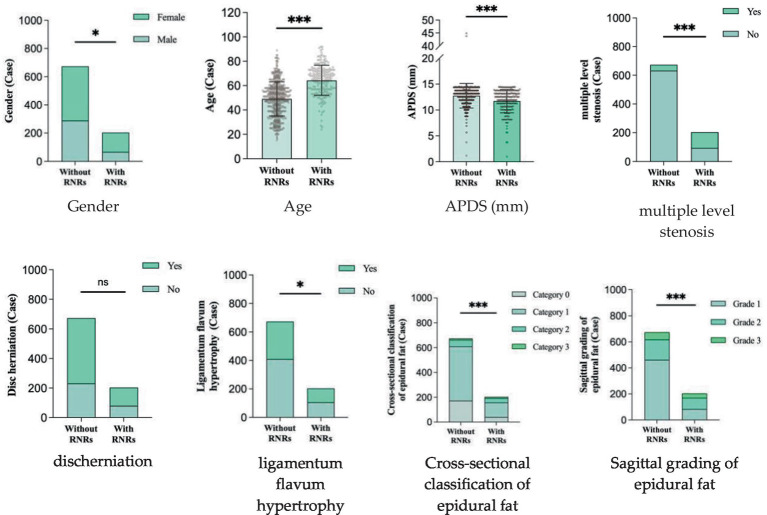
Stacked histograms of single factor analyses of LSS combined RNRs.

The relationship between sex, age, APDS measurements, multiple level stenosis, disc herniation, thickening of the ligamentum flavum, epidural fat cross-sectional classification and sagittal grading and the occurrence of RNRs was found. Compared with the patients who did not have RNRs, most the patients with RNRs were female, older, had smaller APDS measurements, and had both multiple level stenosis and thickening of the ligamentum flavum (*p* < 0.05) (Figure 7); moreover, the distribution of the epidural fat cross-sectional classification and sagittal grading of the two groups was also statistically significant (*p* < 0.05), while the distribution of herniation in the two groups was not statistically significant (*p* > 0.05). For details, see Table 1 and Figure 8.

**Table 1:** One-way analysis of variance of RNRs^a^ (*n* = 878).

Variant	Total ( $\bar{x}\pm s$ )/*n* (%)	Without-RNRs ( $\bar{x}\pm s$ )/*n* (%)	With RNRs ( $\bar{x}\pm s$ )/*n* (%)	t/ $\chi^{2}$ /Z value	*p* value
Sex				6.542	0.011
Male	356 (40.5%)	289 (42.9%)	67 (32.8%)		
Female	522 (59.5%)	385 (57.1%)	137 (67.2%)		
Age	52.61 ± 15.165	49.06 ± 14.098	64.34 ± 12.426	−14.905	< 0.001
APDS^b^ (mm)	12.51 ± 2.393	12.75 ± 2.382	11.69 ± 2.246	−5.939	< 0.001
Multiple level stenosis				247.839	< 0.001
No	728 (82.9%)	633 (93.9%)	95 (46.6%)		
Yes	150 (17.1%)	41 (6.1%)	109 (53.4%)		
Disc herniation				1.571	0.210
Yes	566 (64.5%)	442 (65.6%)	124 (60.8%)		
No	312 (35.5%)	232 (34.4%)	80 (39.2%)		
Ligamentum flavum hypertrophy				5.945	0.024
Yes	362 (41.2%)	264 (39.2%)	98 (48.0%)		
No	516 (58.8%)	410 (60.8%)	106 (52.0%)		
Cross-sectional classification of epidural fat				−3.766	< 0.001
0	214 (24.4%)	173 (25.7%)	41 (20.1%)		
1	554 (63.1%)	437 (64.8%)	117 (57.4%)		
2	88 (10.0%)	53 (7.9%)	35 (17.2%)		
3	22 (2.5%)	11 (1.6%)	11 (5.4%)		
Sagittal grading of epidural fat				−7.017	< 0.001
1	546 (62.2%)	462 (68.5%)	84 (41.2%)		
2	243 (27.7%)	157 (23.3%)	86 (42.2%)		
3	89 (10.1%)	55 (8.2%)	34 (16.7%)		

* *p* value ≤ 0.05 is statistically significant.

a RNRs, redundant nerve roots.

b APDS, anteroposterior diameter of spinal canal.

### 3.2 Relationship between increased epidural fat and redundant nerve roots (LSS)

To further explore whether increased epidural fat is related to LSS combined with RNRs, patients without disc herniation and without ligamentum flavum hypertrophy in this study were selected for inclusion in the next step of the analysis, and it can be assumed that, in these patients, the sagittal plane classifications of epidural fat were distributed differently between the redundant and nonredundant groups (*p* < 0.001), and it cannot be assumed that the epidural fat cross-sectional classification was distributed differently between groups (*p* = 0.054) (see Table 2).

**Table 2:** Distribution of patients.

Variant	Total ( $\bar{x}\pm s$ )/*n* (%)	Without-RNRs^a^ ( $\bar{x}\pm s$ )/*n* (%)	With RNRs ( $\bar{x}\pm s$ )/*n* (%)	*Z* value	*p* value
Cross-sectional classification of epidural fat				1.928	0.054
0	69	57 (82.6%)	12 (17.4%)		
1	105	85 (81.0%)	20 (19.0%)		
2	14	8 (57.1%)	6 (42.9%)		
3	5	2 (40.0%)	3 (60.0%)		
Sagittal grading of epidural fat				3.587	<0.001*
1	142	121 (85.2%)	21 (14.8%)		
2	40	24 (60.0%)	16 (40.0%)		
3	11	7 (63.7%)	4 (36.4%)		

*p value ≤ 0.05 is statistically significant.

a RNRs, redundant nerve roots.

**Figure 9:**
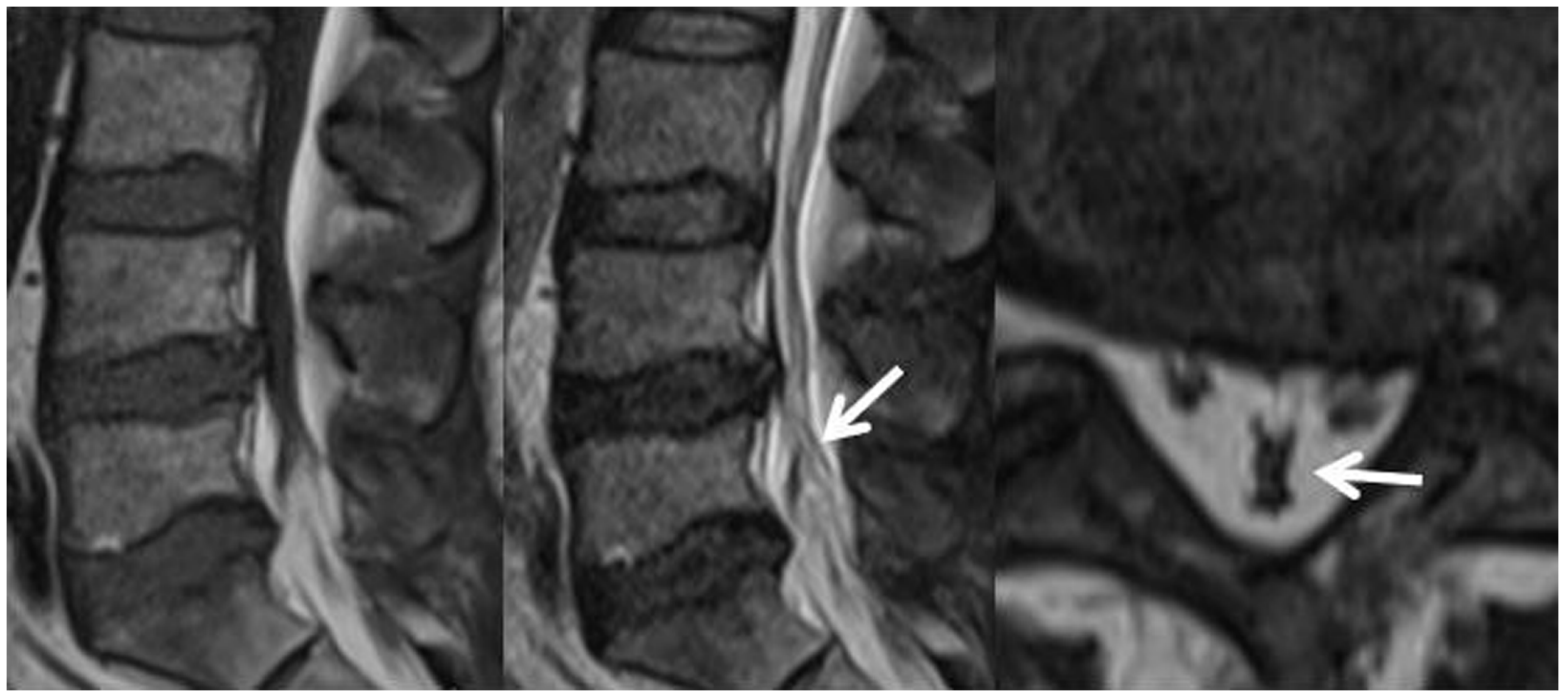
Patient, male, 54 years old, with lumbosacral distension, lumbar 3-sacral 1 paravertebral and interspinous tenderness, epidural fat sagittal grading of grade III, and varying degrees of indentation at the posterior edge of the dural sac, the Y-shaped sign(+), with the cauda equina showing dilated, curved and entangled in the sagittal plane of T2WI.

Further grouping of patients according to whether the dural sac was deformed by pressure as calculated by dichotomous data, it can be assumed that among this group of patients, whether the dural sac was deformed by pressure or not was statistically significant between the two groups and that the proportion of patients with deformation of the dural sac by pressure was greater in the redundancy group (*p* < 0.01), as shown in Table 3 and Figure 9.

**Table 3:** Distribution of patients.

Variant	Total ( $\bar{x}\pm s$ )/*n* (%)	Without-RNRs^a^ ( $\bar{x}\pm s$ )/*n* (%)	With RNRs ( $\bar{x}\pm s$ )/*n* (%)	$\chi^{2}$ -value	*p* value
Classification of epidural fat				6.952	0.008
Group C (category 0 + 1)	174	142 (81.6%)	32 (18.4%)		
Group D (category 2 + 3)	19	10 (52.6%)	9 (47.4%)		
Sagittal grading of epidural fat				13.382	< 0.001
Group A (Grade1)	142	121 (85.2%)	21 (14.8%)		
Group B (Grade2 + 3)	51	31 (60.8%)	20 (39.2%)		

* *p* value ≤ 0.05 is statistically significant.

a RNRs, redundant nerve roots.

### 3.3 Multifactorial analysis

The binary logistic regression analysis was performed using the forward stepwise method with whether LSS patients had combined RNRs (no redundancy = 0, redundancy = 1) as the dependent variable and the statistically significant factors from the univariate analysis as the independent variables (see Table 4 for the assignment method). The final model likelihood ratio χ^2^ = 369.636, df = 5, P0.001, suggesting that the model was generally meaningful. In terms of model fit, the Hosmer–Lemeshow goodness-of-fit test = 3.915, df = 8, *p* = 0.865, suggesting that the model fits well.

**Table 4:** Description of assignments for logistic regression.

Variant	Description of the assignment
Sex	Male = 0, Female = 1
Age	Actual value
APDS^a^ (mm)	Actual value
Multidimensional stenosis	no = 0, yes = 1
Ligamentum flavum hypertrophy	no = 0, yes = 1
Cross-sectional classification-Increased epidural fat	no = 0, yes = 1
Sagittal plane grading-Increased epidural fat	no = 0, yes = 1

a APDS, anteroposterior diameter of spinal canal.

Specific results showed that the older the patient, the smaller the APDS measurements, the higher the multiple level stenosis, and the higher the cross-sectional grading of epidural fat and the sagittal classification, the more likely the occurrence of redundant nerve roots (LSS) (*p* < 0.05). Multiple level stenosis and increased epidural fat were independent risk factors for the occurrence of RNRs, as detailed in Table 5.

**Table 5:** Results of binary logistic regression analyses for the occurrence of LSS^a^ combined with RNRs^b^.

	Logistic regression analysis			ROC^c^ analysis		
	OR^d^	95% CI^e^	*p* value	AUC^f^	Sensitivity (%)	Specificity (%)
Age	1.080	(1.062, 1.099)	< 0.001*	0.793	0.629	0.583
APDS^g^ (mm)	0.816	(0.729, 0.913)	< 0.001*	0.637	0.448	0.666
Multiple level stenosis	14.287	(8.699, 23.464)	< 0.001*	0.737	0.511	0.646
Cross-sectional classification-Increased epidural fat	2.775	(1.828, 4.212)	< 0.001*	0.565	0.408	0.635
Sagittal plane grading-Increased epidural fat	3.113	(2.254, 4.299)	< 0.001*	0.637	0.529	0.562
Combined indicators	–	–	–	0.887	0.782	0.660

*p value ≤ 0.05 is statistically significant.

a LSS, central lumbar spinal stenosis.

b RNRs, redundant nerve roots.

c ROC, receiver operating characteristic.

d OR, odds ration.

e CI, confidence interval.

f AUC, area under the curve.

g APDS, anteroposterior diameter of spinal canal.

**Figure 10:**
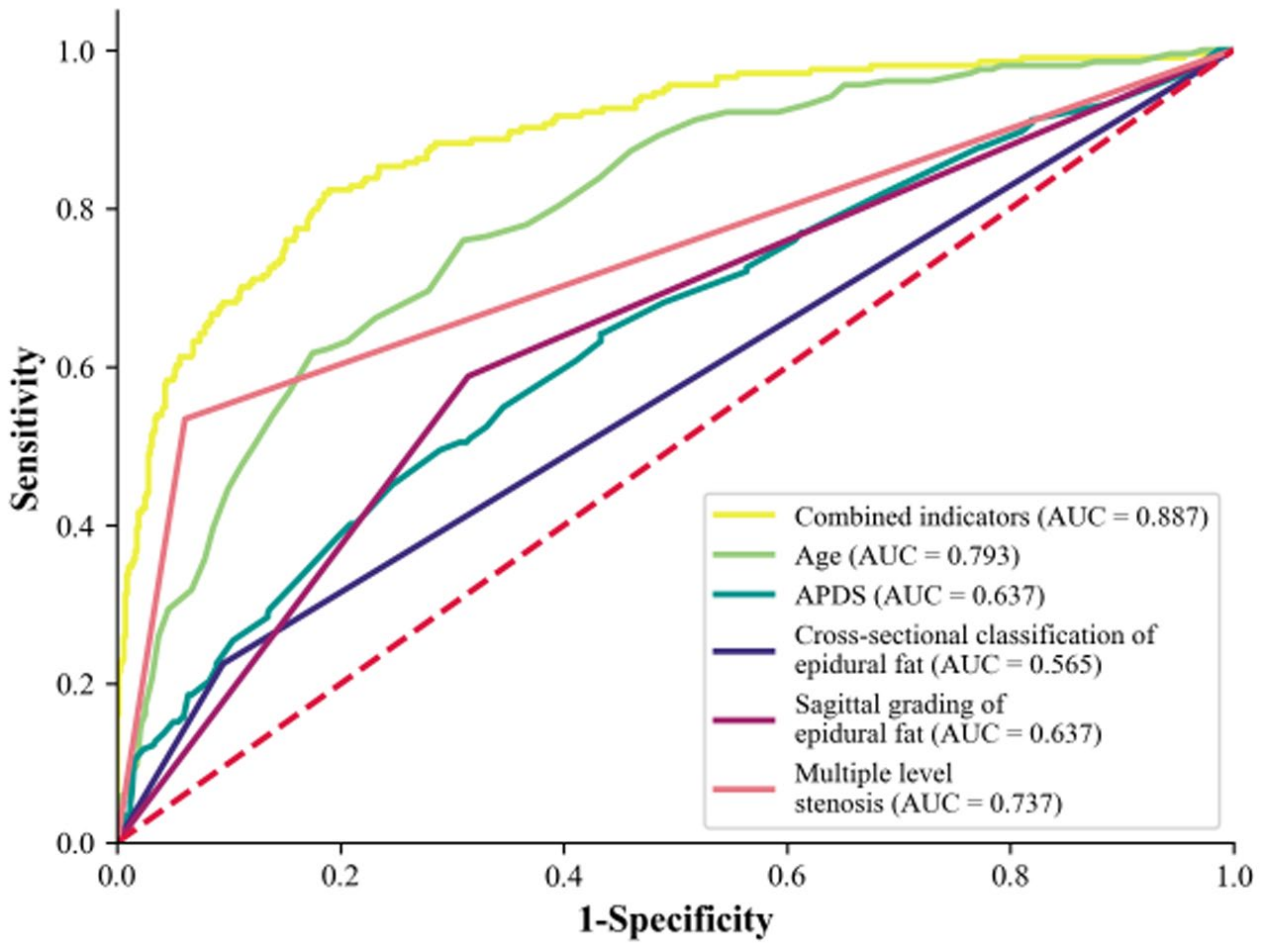
Predicted ROC curves for LSS combined with RNRs.

The predictive effects of age, APDS, multiple level stenosis, and increased epidural fat on LSS combined with RNRs are shown in Table 5. In the ROC analysis, age predicted the incidence of LSS combined with RNRs with an area under the curve (AUC) of 0.793, APDS predicted the incidence of LSS combined with RNRs with an AUC of 0.637, multiple level stenosis predicted the incidence of LSS combined with LSS combined with RNRs with an AUC of 0.737, and the AUC was 0.565 for epidural lipoatrophy in cross-sectional classification for predicting the occurrence of LSS combined with RNRs, and the AUC was 0.639 for epidural capsular lipoatrophy in sagittal classification for predicting the occurrence of LSS combined with RNRs. The AUC of combined age, APDS, multiple level stenosis, and epidural lipoatrophy predicting the occurrence of LSS combined with RNRs improved to 0.887, 95% CI: 0.861 to 0.914, with a sensitivity of 78.2% and a specificity of 66.0%, as shown in Figure 10.

## 4 Discussion

The cause of the formation of RNRs is unclear, and the existing doctrines are mainly composed of including the mechanical pressure doctrine (21) and the microcirculatory disorders doctrine (22, 23). The mechanical pressure theory suggests that in patients with LSS, direct pressure changes lead to compression of the cauda equina, resulting in various clinical symptoms. The microcirculatory impairment theory suggests that lumbar spinal stenosis leads to abnormal functioning of the cauda equina microvasculature, and the cauda equina becomes tortuous and deformed due to ischemia, which leads to the development of RNRs. In our study, we found that there were more female patients in the combined redundant group of LSS than in the nonredundant group, and the older the patient, the smaller the APDS measurements with multiple level stenosis (*p* < 0.05), which may be because degenerative changes in the lumbar vertebrae and the severity of stenosis worsen with age and that the redundant nerve roots (LSS) are subjected to long-term sustained compression and are repeatedly stretched by this action, leading to the development of RNRs. This is consistent with previous reports (6, 13). At the same time, the results also showed that the ADPS values of the redundant group were smaller than those of the non-redundant group, and this difference was statistically significant. This method of measuring the anterior and posterior diameter values to reflect the condition of nerves within the spinal canal also meets the requirements of clinical spinal decompression procedures, such as the lumbar spinous process-splitting laminectomy (LSPSL) (24, 25) and modified LSPSL (26). Unlike traditional laminectomy procedures, these two types of surgeries involve splitting the spinous process into two halves longitudinally in order to reduce damage to ligaments while performing decompression, preserve bone attached to adjacent ligaments, and improve postoperative stability of small joints. As these new surgical techniques are gradually being promoted in clinical practice, preoperative assessment by surgeons regarding distances from inner edges of spinous processes to vertebral plates – which is also measured in this study as anterior and posterior diameters of the spinal canal – will receive more attention.

Moreover, magnetic resonance imaging of the vertebral body and surrounding tissue structures of patients with LSS combined with RNRs was comprehensively evaluated in this study. It was found that disc herniation did not differ between the groups with or without LSS combined with RNRs (*p* > 0.05), and ligamentum flavum hypertrophy differed from that between the groups with or without LSS combined with RNRs (*p* < 0.05), which indicated that ligamentum flavum hypertrophy tended to be concomitant in the group with LSS combined with RNRs. This is also in agreement with the findings of Hur et al. (5), who concluded that disc herniation evokes immediate severe low back pain or radiculopathy (usually IVD protrusion evoked immediate severe low back pain or radiculopathy) and does not cause prolonged compression of the cauda equina root. Therefore, we believe that when the herniated disc causes lateral saphenous fossa stenosis to directly compress the corresponding regional traveling nerves and causes nerve root compression symptoms to appear early, medical treatment can be sought early, and the compression of the spinal canal can be relieved in a timely manner, which does not lead to the occurrence of RNRs; when the herniated disc does not cause lateral saphenous fossa stenosis, i.e., there is no direct compression of the corresponding regional traveling nerves, the narrowing of the canal caused by the protrusion and the change in pressure is homogeneous, a sustained manifestation, and it will cause the affected cauda equina bundle to be pulled repeatedly, leading to the occurrence of RNRs, so RNRS may or may not be present in patients attending the clinic with LSS caused by disc herniation. There was no difference between the groups. Several studies (27–29) have shown that ligamentum flavum hypertrophy is the main causative factor of central spinal stenosis and is involved in long-term chronic compression of the dural sac (5, 30). RNRs due to ligamentum flavum hypertrophy-induced spinal stenosis are a chronic process with a high incidence of RNRs.

Additionally, in this study, we demonstrated that increased spinal epidural fat was also significantly different between the groups of patients with LSS with and without combined RNRs, and both cross-sectional classification and sagittal grading of epidural fat were significantly different between the groups (*p* < 0.05). That is, the LSS combined with RNR group tended to have a concomitant increase in epidural fat (EF). Epidural fat has a high signal on T1-weighted images and a medium signal on T2-weighted images; it is normally deposited to varying degrees in the spinal canal and travels along the spinal canal in a continuous pattern around the dural sac to maintain normal tissue movement and function. Cross-sectional classification and sagittal grading can show the presence or absence of epidural fat on the dural sac and the severity of compression. On magnetic resonance imaging, the normal dural sac appears as a smooth, rounded convex shape that resembles the posterior aspect of the spine. As the amount of epidural fat increases, the posterior edge of the dural sac gradually flattens out, becomes concave, and even forms a Y-shape when the dural sac is compressed in all directions (shown in Figure 5). In our data, there were two cases of LSS in which the increase in epidural fat led to a “Y” sign in the dural sac, and both of them showed RNRs. Magnetic resonance imaging in the sagittal plane was able to show different degrees of epidural fat deposition in the longitudinal direction. While the normal pattern is a smooth and discontinuous distribution of fat of uneven thickness, the increased epidural fat projects toward the dural sac and compresses the posterior edge of the dural sac, resulting in different degrees of indentation. Further analysis of the effect of epidural fat on the pressure deformation of the dural sac on the combined RNRs of LSS showed that the occurrence of combined RNRs of LSS was different between Groups A (Grade 0 + Grade 1) without pressure deformation and B (Grade 2 + Grade 3) with pressure deformation in the transverse plane and between Group C (Grade 1) without pressure deformation and Group D (Grade 2 + Grade 3) with pressure deformation in the sagittal plane. Were all different and statistically significant (*p* < 0.05), and both evaluations of epidural bursa fat showed that increased epidural fat was associated with the occurrence of LSS combined with RNRs (*p* < 0.01), i.e., the distribution of epidural fat could be accurately observed in both positions, and the fat projecting into the dural sac leading to its compression deformation suggested the possible occurrence of RNRs. It was concluded that the increase in epidural bursa fat was a key factor in the major reason for the occurrence of combined RNRs in LSS. Because epidural fat deposits are limited by the right and left bony and ligamentous restrictions, they are more likely to project into the dural sac and exert a constant compressive force on the dorsal side of the dural sac, and this compressive force from the posterior side can act directly on the nerve roots, i.e., the cauda equina fasciculus, which is deposited to the dorsal portion of the dural sac due to gravity, resulting in RNRs. These findings highlight the importance of peridural fat in the development of RNRs and provide strong support for a precise and comprehensive diagnosis of LSS combined with RNRs.

On this basis, we explored the main pathophysiological factors and anatomical predictions for the occurrence of LSS combined with RNRs. It was shown that among the main factors contributing to the occurrence of RNRs, the abnormal function of microvessels from any cause, resulting in ischemia and hypoxia of the cauda equina, which in turn leads to neurocirculatory and trophic deficits, will gradually lead to tortuous and encircling cauda equina, which shows that microcirculatory deficits are crucial in the occurrence of RNRs. Our results showed that patient age, APDS, multiple level stenosis, and increased epidural fat were independent risk factors for the formation of LSS combined with RNRs in the included patients. This may be due to the development of atherosclerosis in older patients. The smaller the APDS measurement, the higher the degree of stenosis, and the greater the degree of compression on the dural sac, the more likely that RNRs will occur in patients with LSS, whereas sex and ligamentum flavum hypertrophy affected whether LSS was combined with RNRs in the univariate analyses but were excluded from multivariate analyses, considering that because sex does not manifest itself clearly in the vascular differences of individual microcirculation and the ligamentum flavum hypertrophy is only restricted to the posterior lateral wall of the spinal canal, often with myofibrillar hypertrophy, and often with less impact on the cauda equina microcirculation without peripheral vascular access. The thickening of the ligamentum flavum is limited to the posterior lateral wall of the spinal canal, often with hypertrophy of the muscle belly, the compression of the dural sac is relatively limited without peripheral vascular access, and the effect on the microcirculation of the cauda equina is also small, often manifesting itself as a predominantly mechanical pressure on the dural sac. After multifactorial logistic regression analysis, the OR values of increased epidural fat in the transverse and sagittal planes were 2.775 and 3.113, respectively, and both methods suggested that the risk of combined occurrence of RNRs in patients with LSS was significantly higher when there was an increase in epidural fat. The possible reason is that the trophoblastic vessels of the cauda equina travel in the epidural fat with microscopic small branches, and the increased adipose tissue can directly affect these microvessels, which can lead to circulatory and trophic disorders of the cauda equina, and the cauda equina gradually becomes tortuous and encircling; the deposition of epidural fat often occurs in multilayered and multisegmental ways, and there is a superimposed effect on the cauda equina in terms of both the mechanical stresses and microcirculatory effects. This result is also validated by the association of multiple level stenosis with the occurrence of LSS combined with RNRs. These findings emphasize the importance of increased epidural fat in the mechanism of the development of RNRs in patients with LSS.

ROC curve analysis showed that age, APDS value, multiple level stenosis, epidural fat cross-sectional classification and sagittal grading had predictive ability for the occurrence of RNRs. Logistic regression analysis yielded a composite metric AUC of 0.887 with a sensitivity of approximately 0.775 and specificity of approximately 0.675. The combination of multiple risk factors can further improve the predictive performance and provide an objective imaging reference for disease monitoring and treatment plan selection or evaluation.

Our study has several limitations. First, the limited sample size and numerous LSS parameters may have affected the accuracy of multivariate logistic regression. Second, the relationship between LSS parameters and the degree of stenosis was not investigated in this study. There is a need to further investigate the correlation between RNRs and the performance of surrounding tissue structures at different degrees of LSS. Third, it is unclear whether peripheral tissue structure parameters can predict recovery or progression after LSS combined with RNRs due to the lack of appropriate follow-up information.

## 5 Conclusion

Female patients, older age, smaller median sagittal diameter of the dural sac, ligamentum flavum hypertrophy, and increased epidural fat are the main pathophysiological bases for the development of redundant nerve roots (LSS) in the cauda equina; among them, age, multiple level stenosis, and increased epidural fat can be used to predict and monitor lumbar stenosis complicating the redundant nerve root (LSS) sign.

## Data availability statement

The raw data supporting the conclusions of this article will be made available by the authors, without undue reservation.

## Ethics statement

The studies involving humans were approved by the Medical Ethics Committee of the People’s Hospital of Liangjing New Area, Chongqing. The studies were conducted in accordance with the local legislation and institutional requirements. Written informed consent for participation was not required from the participants or the participants’ legal guardians/next of kin due to the observational nature of the study.

## Author contributions

JQ: Conceptualization, Formal analysis, Investigation, Methodology, Project administration, Validation, Writing – original draft, Writing – review & editing. KL: Data curation, Software, Validation, Visualization, Writing – review & editing. XL: Data curation, Writing – review & editing. CY: Conceptualization, Formal analysis, Project administration, Resources, Supervision, Writing – review & editing, Writing – original draft.
